# Efficacy, effectiveness, and behavior change trials in exercise research

**DOI:** 10.1186/1479-5868-7-81

**Published:** 2010-11-12

**Authors:** Kerry S Courneya

**Affiliations:** 1 Faculty of Physical Education and Recreation, University of Alberta, Edmonton, Alberta, Canada

## Abstract

**Background:**

The widespread incorporation of behavioral support interventions into exercise trials has sometimes caused confusion concerning the primary purpose of a trial. The purpose of the present paper is to offer some conceptual and methodological distinctions among three types of exercise trials with a view towards improving their design, conduct, reporting, and interpretation.

**Discussion:**

Exercise trials can be divided into "health outcome trials" or "behavior change trials" based on their primary outcome. Health outcome trials can be further divided into efficacy and effectiveness trials based on their potential for dissemination into practice. Exercise efficacy trials may achieve high levels of exercise adherence by supervising the exercise over a short intervention period ("traditional" exercise efficacy trials) or by the adoption of an extensive behavioral support intervention designed to accommodate unsupervised exercise and/or an extended intervention period ("contemporary" exercise efficacy trials). Exercise effectiveness trials may emanate from the desire to test exercise interventions with proven efficacy ("traditional" exercise effectiveness trials) or the desire to test behavioral support interventions with proven feasibility ("contemporary" exercise effectiveness trials). Efficacy, effectiveness, and behavior change trials often differ in terms of their primary and secondary outcomes, theoretical models adopted, selection of participants, nature of the exercise and comparison interventions, nature of the behavioral support intervention, sample size calculation, and interpretation of trial results.

**Summary:**

Exercise researchers are encouraged to clarify the primary purpose of their trial to facilitate its design, conduct, and interpretation.

## 

Randomized controlled trials (RCTs) are a common design for testing many health interventions including exercise. In recent years, exercise RCTs have become more complex and challenging because of a greater focus on methodological rigor and the desire to test longer exercise interventions, unsupervised exercise interventions, and higher volume exercise interventions. These changes have necessitated a greater focus on exercise adherence in these trials and have stimulated the incorporation of behavioral support interventions into many exercise trials for which behavior change was not the primary purpose. Behavioral support interventions consist of strategies to improve exercise adherence such as incentives, print materials, telephone counselling, group sessions, websites, and other behavior modification techniques. The incorporation of behavioral support interventions into exercise RCTs has been a major methodological advance in exercise research, however, it has sometimes caused confusion concerning the primary purpose of a trial. Was the trial designed to improve a health outcome or to understand exercise behavior change? Was the trial designed to demonstrate efficacy or effectiveness? This confusion may lead to improperly designed trials and/or the misinterpretation of trial results. The purpose of the present paper is to offer some conceptual and methodological distinctions among different types of exercise trials with a view towards improving their design, conduct, reporting, and interpretation.

## Health Outcome Trials Versus Behavior Change Trials

**Figure 1:**
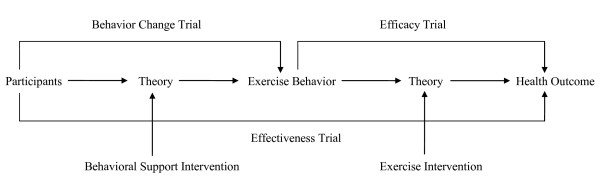
**Schematic representation of three types of exercise trials**.

In simplest terms, exercise RCTs can be divided into "health outcome trials" or "behavior change trials" (Figure 1). Health outcome trials are those trials in which the primary purpose is to examine the effects of an exercise intervention on some health outcome such as cardiorespiratory fitness, body composition, psychosocial functioning, biomarkers, or disease states. Behavior change trials, on the other hand, are those trials in which the primary purpose is to examine the effects of a behavioral support intervention on some aspect of exercise behavior itself such as the type, volume, intensity, or maintenance of exercise. In the past, these two types of exercise trials largely led separate lives and were viewed as independent but related steps towards improving health through exercise. In recent years, however, several major changes in the exercise field have brought these two types of trials together.

## The Rise of Behavioral Support Interventions in Health Outcome Trials

Several key changes have occurred in the exercise field over the past two decades that have prompted the inclusion of behavioral support interventions into many health outcome trials: (a) the adoption of the Consolidated Standards of Reporting Trials (CONSORT) [1], (b) longer exercise intervention trials, (c) unsupervised exercise intervention trials, and (d) higher volume exercise intervention trials. Prior to the CONSORT guidelines [1], exercise health outcome trials rarely incorporated behavioral support interventions into their methodology, probably because of the exclusively supervised exercise protocols, the relatively short exercise interventions (e.g., 12-24 weeks), and methodological practices that were accepted at the time. In pre-CONSORT exercise health outcome trials, participants who did not adhere to the exercise intervention were often removed from the analyses and/or not reported in the trial. The solution to nonadherence in these trials was to recruit more participants until the desired sample size of "adherers" was achieved. The acceptability of this practice was brought into question with the adoption of the CONSORT guidelines [1] by most medical, health, and behavioral journals, including one of the leading exercise science journals, *Medicine & Science in Sports & Exercise*. Among its many recommendations for improving the quality of RCTs, the CONSORT guidelines stated that all trial participants must be analyzed according to their assigned condition regardless of their level of adherence to the trial protocol. Consequently, nonadherence became a potentially important issue even in health outcome trials with supervised exercise protocols and shorter length interventions.

At the same time, researchers began conducting longer exercise interventions (e.g., 1-2 years) to determine the sustained effects of exercise over time and also to examine more distal effects of exercise such as disease endpoints. The expansion of health outcome trials to one year, two years, and even longer provided further impetus for the inclusion of behavioral support interventions as it was obvious that sustained exercise adherence would be a challenge even with motivated participants and supervised exercise protocols. Moreover, other researchers began testing unsupervised exercise interventions with the goal of improving the potential for translation of exercise research into practice. In these trials, it was also clear that adherence to unsupervised exercise would require some level of behavioral support, even for shorter length interventions. Finally, researchers became interested in testing higher volumes of exercise that were hypothesized to be necessary for addressing some of the more intractable health outcomes (e.g., obesity, disease endpoints). Again, it was clear that the testing of these higher exercise volumes (e.g., 150-300 minutes per week) would require significant behavioral support. These four factors-adoption of the CONSORT guidelines [1], longer exercise interventions, unsupervised exercise interventions, and higher volume exercise interventions-prompted the incorporation of behavioral support interventions into many health outcome trials for which behavior change was not the primary purpose.

## Efficacy (Explanatory) Versus Effectiveness (Pragmatic) Trials

Complicating the matter further is the growing importance of the distinction between efficacy and effectiveness trials [2]. Efficacy trials (also called explanatory trials) determine whether an intervention produces the intended effect under ideal circumstances whereas effectiveness trials (also called pragmatic trials) determine whether an intervention produces the intended effect under "real-world" conditions [3]. Most RCTs, including exercise, contain a mix of efficacy and effectiveness elements [4]. In this sense, efficacy and effectiveness trials exist along a continuum and their designation is a matter of degree. Moreover, there are many individual features of an RCT and each can vary along the efficacy-effectiveness continuum; not just the overall RCT [2,5-8]. One of the key differences between efficacy and effectiveness trials from this paper's perspective is the anticipated level of adherence to the intervention. Reflecting this key issue, efficacy refers to whether an intervention works in people who receive it (i.e., who adhered to the protocol) whereas effectiveness refers to whether an intervention works in people to whom it has been offered (i.e., it assumes at least some level of nonadherence) [4]. The incorporation of behavioral support interventions into many exercise health outcome trials has further clouded the complex distinction between efficacy and effectiveness trials in exercise research.

## Traditional and Contemporary Efficacy Trials in Exercise Research

In exercise research, a distinction might be made between "traditional" exercise efficacy trials and "contemporary" exercise efficacy trials. In traditional exercise efficacy trials, exercise adherence was typically assured by a completely supervised exercise intervention testing a relatively modest exercise volume (e.g., 3 days per week for 30 minutes) over a relatively short intervention (e.g., 12-24 weeks). In one sense, the supervised exercise itself was the "behavioral support intervention" that ensured the high level of exercise adherence over the short intervention period (i.e., optimal conditions). As such, there was relatively little need to incorporate any modern behavioral support techniques into traditional exercise efficacy trials. As noted earlier, the adoption of the CONSORT guidelines [1] may make behavioral support interventions potentially important even for these traditional exercise efficacy trials.

**Figure 2:**
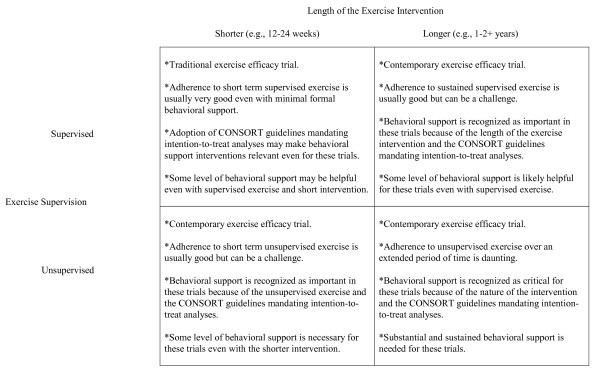
**Level of behavioral support needed for exercise efficacy trials based on the degree of exercise supervision and length of the exercise intervention**.

Contemporary exercise efficacy trials, on the other hand, typically include one or more of the following features: (a) an unsupervised or partially supervised exercise intervention, (b) an extended exercise intervention (e.g., 1-2+ years), and/or (c) a relatively large volume of exercise (e.g., 150-300 minutes of exercise per week). The nature of these trials makes it unlikely that they are able achieve a high level of adherence sufficient to test the efficacy of the exercise intervention without including some level of behavioral support. In contemporary exercise efficacy trials, the goal of the behavioral support intervention is to obtain a high level of exercise adherence over an extended period of time so that the primary question concerning the particular health outcome can be answered. The behavioral support intervention, in essence, is viewed as the primary mechanism for ensuring a high level exercise adherence in the absence of completely supervised exercise over a short intervention period. The level of behavioral support needed in contemporary exercise efficacy trials likely varies by the degree of exercise supervision, the length of the exercise intervention, and the volume of the exercise intervention (Figure 2).

## Traditional and Contemporary Effectiveness Trials in Exercise Research

Exercise efficacy trials and effectiveness trials are both health outcome trials with the primary purpose of determining the effects of an exercise intervention on a health outcome. Contemporary exercise efficacy trials and effectiveness trials also both include modern behavioral support interventions to help answer the primary health outcome question. Consequently, the distinguishing feature between contemporary exercise efficacy trials and effectiveness trials appears to be the nature of the behavioral support intervention. In contemporary exercise efficacy trials, researchers typically develop or adopt a behavioral support intervention that is intensive enough to achieve a high level of adherence to the exercise intervention over an extended period of time so that the primary health outcome question can be answered. Efficacy trials, by definition, are less concerned about whether the behavioral support intervention is feasible in practice. Conversely, an exercise effectiveness trial typically adopts a behavioral support intervention that is consistent with practice or at least deemed potentially feasible to put into practice. The goal is then to determine whether the exercise intervention achieved by a feasible behavioral support intervention is sufficient for improving some important health outcome.

There also appears to be two approaches to effectiveness trials in exercise research. The "traditional" approach to an effectiveness trial is usually one that proceeds from a successful efficacy trial demonstrating the benefits of an exercise intervention under optimal conditions. The purpose of the traditional effectiveness trial is then to determine if the benefits of the exercise intervention are still evident under "real-world" conditions (i.e., in practice). The "contemporary" approach to an effectiveness trial, on the other hand, is usually one that proceeds from a successful behavior change trial demonstrating that a feasible behavioral support intervention is able to increase exercise behavior to a potentially meaningful level. The purpose of the contemporary effectiveness trial is then to determine if the exercise intervention achieved by the feasible behavioral support intervention is sufficient for improving some health outcome in practice.

## Efficacy, Effectiveness, and Behavior Change Trials in Exercise Research

As noted earlier, the widespread adoption of behavioral support interventions into many exercise RCTs has sometimes caused confusion concerning the primary intent of the trial. At the simplest level, it is sometimes unclear if the intent was to conduct a health outcome trial with behavioral support or a behavior change trial with secondary health outcomes. At a more complex level, it is sometimes unclear if the intent was to conduct an efficacy trial but an inadequate level of behavioral support resulted in modest adherence and gave the impression of an effectiveness trial. In other cases, it is unclear if the intent was to conduct an effectiveness trial but an inadequate level of behavioral support resulted in no improvements in health outcomes and gave the impression of a behavior change trial. Still, in other cases, it is unclear if the intent was to conduct a behavior change trial but improvements in some health outcomes gave the impression of an effectiveness trial. In the following sections, some potentially distinguishing features among efficacy, effectiveness, and behavior change trials are discussed under the headings of selection of primary and secondary outcomes, adoption of theoretical models, selection of participants, nature of the exercise intervention, nature of the behavioral support intervention, nature of the comparison intervention, sample size calculation and analysis, and interpretation of trial results (Table 1).

**Table 1:** Summary of some distinguishing features among efficacy, effectiveness, and behavior change exercise trials.

	Efficacy Trial			
			
	Traditional	Contemporary	Effectiveness Trial	Behavior Change Trial
Primary Outcome:	A health outcome	A health outcome	A health outcome	An exercise behavior outcome
Secondary Outcomes:	Other health outcomes	Other health outcomes	Other health outcomes	Other behavior/health outcomes
Protocol Deviations:	Not exercising	Not exercising/behavioral support	Not exercising/behavioral support	Not completing behavioral support
				
Theoretical Model:	Outcome model needed	Outcome model needed	Outcome model needed	Outcome model not needed
	Behavior model not needed	Behavior model beneficial	Behavior model needed	Behavior model needed
				
Participants:	Highly selected	Highly selected	Moderately selected	Variably selected
	Not exercising/limited exercise	Not exercising/limited exercise	Exercise levels reflective of practice	Variable exercise levels
	Lower generalizability	Lower generalizability	Higher generalizability	Higher generalizability
				
Exercise Intervention:	Completely supervised	Completely/partially supervised	Unsupervised/partially supervised	NA (exercise is the outcome)
	Precise exercise type/volume	Partially flexible types/volumes	Flexible exercise types/volumes	Flexible exercise types/volumes
	Agree to exercise	Agree to exercise	Agree to exercise	Asked to exercise
	No/limited behavioral support	Agree to behavioral support	Agree to behavioral support	Agree to behavioral support
				
Behavioral Support Intervention:	Minimal/informal	Extensive/comprehensive	Moderate/feasible for dissemination	Varied but systematic and feasible
				
Comparison Intervention:	No exercise intervention	Minimal exercise intervention	Standard of care	At least exercise recommendation
	Agree not to exercise	Allowed to exercise	Allowed to exercise	Asked to exercise
	No behavioral support	No/limited behavioral support	Behavioral support as per standard care	Behavior support based on purpose
				
Sample Size:	Powered for health outcome	Powered for health outcome	Powered for health outcome	Powered for behavioral outcome
	No allowance for nonadherence	Allowance for nonadherence	Allowance for nonadherence	No allowance for nonadherence
				
Analysis:	Intention-to-treat	Intention-to-treat	Intention-to-treat	Intention-to-treat
	Prespecified subgroup analyses	Prespecified subgroup analyses	Prespecified subgroup analyses	Prespecified subgroup analyses
				
Interpretation of Results:				
				
Positive trial	Improves primary health outcome	Improves primary health outcome	Improves primary health outcome	Improve primary behavior outcome
	Adequate exercise adherence	Adequate exercise adherence	Adequate exercise adherence	Adequate support adherence
		Support adherence not required	Support adherence not required	
				
Negative trial	No change in health outcome	No change in health outcome	No change in health outcome	No change in behavioral outcome
	Adequate exercise adherence	Adequate exercise adherence	Adequate exercise adherence	Adequate support adherence
		Support adherence not required	Support adherence not required	
				
Inconclusive trial	No change in health outcome	No change in health outcome	No change in health outcome	No change in behavioral outcome
	Inadequate exercise adherence	Inadequate exercise adherence	Inadequate exercise adherence	Inadequate support adherence
				
Nature of the Result:				
				
Definitive	Negative trial	Negative trial	Positive trial	Negative trial
	No effect in practice is likely	No effect in practice is likely	Effect in practice is likely	No effect in practice is likely
				
Ambiguous	Positive trial	Positive trial	Negative trial	Positive trial
	Effect in practice is unknown	Effect in practice is unknown	Effect under better conditions unknown	Effect on health outcomes unknown
				(unless shown in trial or literature)

## Selection of Primary and Secondary Outcomes

Perhaps the most obvious and important distinction between health outcome trials and behavior change trials is the identification of the primary outcome. In efficacy and effectiveness trials, the primary outcome is a health outcome (e.g., aerobic fitness, biomarkers, quality of life, disease outcomes). In behavior change trials, the primary outcome is an aspect of exercise behavior (e.g., type, dose, attendance, number of steps). Although this distinction may appear simple and straight forward, confusion can arise in exercise trials when both behavior and health variables are identified as outcomes, and especially when both are identified as primary outcomes. Behavior change does not appear to be an appropriate primary or secondary outcome in efficacy or effectiveness trials because outcomes in an RCT are defined as events that are present after the participants receive the intervention [4]. Given that exercise is the actual intervention in a health outcome trial, adherence to the intervention itself cannot be an outcome of the intervention. Failure to exercise in a health outcome trial is not a negative effect on a primary or secondary outcome, it is a protocol deviation. The fact that the exercise may be unsupervised does not make it an outcome in an efficacy or effectiveness trial.

Conversely, it does seem acceptable, and even meritorious, to identify health outcomes as secondary outcomes in behavior change trials because these are potential outcomes that may follow from the behavioral support intervention and the ensuing exercise behavior change. If improved, these health outcomes may indicate a particularly meaningful level of behavior change. In this context, the goal is to determine whether the exercise behavior that results from such a behavioral support intervention is sufficient for improving some important health outcome. For the same reasons as above, however, it does not seem appropriate to identify adherence to the behavioral support intervention (e.g., telephone calls completed, attendance at group sessions, visits to the website) as a primary or secondary outcome in a behavior change trial. Failure to complete the behavioral support intervention in a behavior change trial is a protocol deviation.

## Adoption of Theoretical Models

Another important distinction among efficacy, effectiveness, and behavior change trials is the nature of the theories that inform the research. In simplest terms, there are two types of theories or models: theories that explain the outcomes of a behavior and theories that explain the determinants of a behavior (see Figure 1). Outcome theories attempt to explain how the exercise intervention (e.g., type, dose, intensity, frequency) improves a health outcome. These theories or models often include biological or physiological variables but might also include health-related fitness or psychosocial variables depending on the nature of the health outcome. Behavioral theories, on the other hand, attempt to explain how a behavioral support intervention (e.g., goal setting, physician recommendation, telephone counselling) might improve exercise behavior. These theories often focus on social cognitive variables such as attitude, self-efficacy, and social support but might also include a wider range of variables such as physiological and environmental factors.

In efficacy trials, researchers should select an outcome theory that will explain how the exercise intervention is thought to change the health outcome. Traditional efficacy trials involving minimal behavioral support would not normally need to adopt a behavioral theory. Contemporary efficacy trials, however, would likely benefit from following a behavioral theory because of the substantial behavioral support intervention that is often included and the greater risk of nonadherence. In effectiveness trials, it would seem that researchers should adopt both an outcome theory to guide the exercise intervention and a behavioral theory to guide the behavioral support intervention. In behavior change trials, researchers typically need only to adopt a behavioral theory. Confusion can arise in exercise trials when there is a "mismatch" between the type of theory and the reported outcomes. That is, exercise trials should not cite behavioral theories to claim they are "theory-based" when reporting health outcomes and, conversely, they should not cite outcome theories to claim they are "theory-based" if they report on the determinants of exercise adherence.

## Selection of Participants

The selection of participants may also vary across efficacy, effectiveness, and behavior change trials. In efficacy trials, participants are typically selected based on their motivation and ability to complete and respond to the particular intervention [6,8]. In exercise efficacy trials, this usually means selecting participants not currently performing the particular exercise intervention yet capable of performing the exercise intervention. In effectiveness trials, participants are only moderately selected based on their motivation and ability because of the desire to reflect "real-world" conditions that broaden eligibility criteria [6,8]. In exercise effectiveness trials, this may mean including participants currently performing some or even all of the intended exercise intervention if this reflects the target group in clinical or public health practice. In behavior change trials, participants may have various levels of motivation and ability depending on the focus of the trial. Because the goal is not to improve a health outcome per se, a behavior change trial might target people with no interest in exercise, some interest in exercise, or a strong interest in exercise. Moreover, it may include participants currently performing no exercise, some exercise, or already meeting the exercise prescription with the goal of maintaining exercise behavior. Given the differences in participant selection, the generalizability of findings is usually lower in efficacy trials and higher in effectiveness and behavior change trials [6].

Moreover, participants in exercise RCTs should generally be asked to agree to the trial protocol prior to randomization. In traditional efficacy trials, the trial protocol includes supervised exercise. In contemporary efficacy and effectiveness trials, the trial protocol might include supervised and unsupervised exercise as well as the behavioral support intervention. Consequently, participants in health outcome trials should be asked to agree to do (or try to do) the exercise intervention as well as the behavioral support intervention as a method of supporting their exercise adherence. In behavior change trials, the trial protocol includes the behavioral support intervention but not the exercise itself because that is the outcome. Consequently, intervention participants in a behavior change trial should be asked to agree to follow the behavioral support intervention but not to agree to do the exercise because that is the primary outcome of the trial.

## Nature of the Exercise Intervention

The nature of the exercise intervention may also vary among exercise trials [8]. In efficacy trials, there is often a precise exercise intervention for participants to follow that includes the type, frequency, intensity, duration, and progression of exercise. For example, participants may be asked to exercise on a cycle ergometer three times per week for 30 minutes at 70-85% of their maximum capacity. Typically, there is limited flexibility in how participants achieve the exercise intervention. In effectiveness trials, there is often a general exercise intervention for participants to follow (e.g., a desired volume) and there may be considerable flexibility in how participants are allowed to achieve the exercise intervention. For example, participants may be asked to exercise for 10 metabolic equivalent task (MET) hours per week but be allowed to choose any type, frequency, intensity, or duration of exercise that meets this general prescription. Alternatively, participants may receive prespecified ranges of exercise from which to chose (e.g., several types of aerobic exercise, moderate-to-vigorous exercise, 3-5 days/week, 20-60 minutes in duration). Moreover, effectiveness trials often establish only a minimum exercise goal, not a maximum goal (e.g., participants may be asked to perform at least 150 minutes of exercise/week but be allowed to exceed this goal). In behavior change trials, there is no exercise intervention per se because exercise is the outcome. Nevertheless, there is often an exercise behavioral goal that participants are asked to achieve as part of the behavioral support intervention.

## Nature of the Behavioral Support Intervention

Another major distinction among exercise trials is the nature of the behavior support intervention. As noted earlier, in traditional exercise efficacy trials, there is typically no formalized behavioral support intervention. Exercise adherence is usually achieved by highly select participants, the shorter length of the intervention, and the supervised exercise that likely entails at least some positive social support and accountability. In contemporary efficacy trials involving unsupervised exercise or an extended intervention, the behavioral support intervention is usually sophisticated and comprehensive, but it is typically a "kitchen sink" approach. That is, contemporary efficacy trials usually incorporate as many behavioral support techniques and motivational strategies as necessary because behavior change is a means to an end and not an end in itself. The goal of these trials is to determine if a particular exercise intervention improves an important health outcome under optimal conditions, not to systematically disentangle what components of the behavioral support intervention might be helpful or to be concerned about the feasibility of the intervention. In efficacy trials, close monitoring of exercise adherence and behavioral support adherence is required during the trial to ensure corrective action is taken if adherence falters [8].

In effectiveness trials, the behavioral support intervention is usually one that reflects practice or at least has the potential for being translated into practice. Similar to efficacy trials, however, the goal of effectiveness trials is to determine if a particular exercise intervention improves some health outcome, not to systematically disentangle what components of the behavioral support intervention worked. Any monitoring and corrective action for exercise adherence and/or behavioral support adherence in effectiveness trials should be consistent with the intended practice context [8]. Conversely, in behavior change trials, the behavioral support intervention may be simple or sophisticated depending on the question; however, the focus is on systematically evaluating the individual or packaged behavioral support intervention to determine its effects on exercise behavior. There is no ongoing monitoring of exercise behavior in behavior change trials because exercise behavior is the primary outcome. Monitoring and corrective action for the behavioral support intervention may be considered depending on the nature of the trial.

## Nature of the Comparison Intervention

The comparison intervention also differs among efficacy, effectiveness, and behavior change trials. In traditional efficacy trials, participants in the comparison group are often asked not to exercise or not to increase their exercise from baseline and to agree to these instructions prior to randomization. These instructions are optimal for efficacy trials because contamination in the comparison group is as problematic as nonadherence in the intervention group. In efficacy trials, the goal is to determine if the exercise intervention improves a health outcome under optimal conditions. The optimal condition includes 100% adherence in the intervention group and 0% contamination in the comparison group. Asking comparison participants not to exercise is often accepted by research ethics boards depending on the purpose of the study, the length of the intervention, and the risk to participants of not exercising.

The comparison group intervention in contemporary efficacy trials and effectiveness trials is much more complex for methodological and ethical reasons. Asking comparison participants not to exercise or not to increase their exercise may not be considered ethical if it is for an extended period of time or if the population is considered at-risk. It may not be scientifically justifiable in effectiveness trials where the goal is to replicate "real-world" environments where comparison participants are free to exercise if they want. This complexity has led to a great deal of variability in what comparison participants are asked to do in contemporary efficacy and effectiveness trials. In some trials, comparison participants are not asked anything but are given standard health materials that often include information on exercise (e.g., public health exercise guidelines). In other trials, they are explicitly recommended to exercise but are not provided with any of the behavioral support intervention or only a limited version of the intervention (e.g., printed materials only, one behavioral support session, a few telephone calls). Still, in other trials, they are given a more extensive health intervention that may be important for general health or for their specific disease condition but it may or may not include exercise. The lack of clear exercise instructions to the comparison group in contemporary efficacy trials is probably an attempt to balance the ethical concern of having comparison participants not exercise, with the scientific concern of maximizing differences in exercise behavior between the intervention and comparison groups.

In effectiveness trials, participants should receive the current standard of care for exercise if one already exists in the particular practice setting [8]. If not, the lack of clear exercise instructions to comparison participants is probably the correct approach ethically and scientifically because the goal is to determine the effects of exercise in a "real world" setting in which comparison participants are allowed to exercise on their own but may not receive any support.

In behavior change trials, the comparison group intervention has also varied. It does not seem scientifically justifiable, however, to ask comparison participants in a behavior change trial not to exercise or not to increase their exercise; or even not to provide them with any instructions. The utility of a behavioral support intervention that is superior to a comparison group that was asked not to exercise or that was not provided with any instructions to exercise seems dubious. Consequently, except in cases where the recommendation itself is the behavior support intervention under investigation (e.g., physician recommendation, oncologist recommendation), it would seem that comparison groups in behavior change trials, at a minimum, should be recommended the same level of exercise as the intervention group. Any additional behavioral support provided to the comparison group in a behavior change trial (including any contact control) would depend on the nature and purpose of the trial and what the researchers wanted to be able to say about the intervention.

## Sample Size Calculation and Analysis

In traditional efficacy trials, the trial is powered for the primary health outcome and, given that nonadherence or contamination are not anticipated, they are typically not factored into the power calculation. Allowance may be made for loss-to-follow-up, but this is a different issue. In contemporary efficacy and effectiveness trials, the trial is also powered for the primary health outcome, however, an *a priori *allowance for some prespecified level of nonadherence and contamination may be factored into the power calculation. In behavior change trials, the trial should be powered for the primary behavioral outcome and typically no allowance is made for any nonadherence or contamination with the behavioral support intervention. The primary analysis in all trials should follow the intention-to-treat principle [1]. Any secondary analysis that deviate from intention-to-treat should be prespecified [1].

## Interpretation of Trial Results

Accurate interpretation of trial results is important but not always provided [9]. In an efficacy trial, failure to improve the primary health outcome in the face of adequate exercise adherence (however defined) is interpreted as a negative trial (i.e., the exercise intervention does not work for that health outcome). Improvements in important secondary health outcomes may be noteworthy but do not change the overall interpretation of the trial. Poor exercise adherence in an efficacy trial is a major protocol deviation. Failure to improve the primary health outcome in the face of inadequate exercise adherence in an efficacy trial results in an inconclusive trial. An inconclusive trial is one in which the primary question could not be answered because of some substantial methodological problem such as poor protocol adherence. Researchers conducting efficacy trials should resist the temptation of interpreting an inconclusive efficacy trial as a negative effectiveness trial or a positive behavior change trial.

In an effectiveness trial, interpretation of the results is the same as for an efficacy trial. In addition, poor adherence to the behavioral support intervention in an effectiveness trial or contemporary efficacy trial is also a protocol deviation. Nevertheless, even if poor adherence to the behavioral support intervention was the likely cause of the poor exercise adherence, it is the lack of exercise adherence that renders the trial inconclusive, not the poor adherence to the behavioral support intervention. Researchers conducting contemporary efficacy trials or effectiveness trials should resist the temptation of interpreting a negative or inconclusive health outcome trial as a positive behavior change trial.

In a behavior change trial, failure to improve the primary behavioral outcome in the face of adequate adherence to the behavioral support intervention (however defined) is interpreted as a negative trial (i.e., the behavioral support intervention does not work). Even if secondary health outcomes improve, the trial would still be considered negative as these outcomes are unlikely to be attributable to the exercise behavior change itself (assuming accurate measurement of exercise) and, in any case, were not the primary outcome [9]. Conversely, a positive change in the primary behavioral outcome is interpreted as a positive trial even if secondary health outcomes do not improve. Certainly, improvements in secondary health outcomes would further substantiate the importance of the behavior change achieved in the trial, but they were not the primary focus. In a behavior change trial, failure to improve the primary behavioral outcome in the face of inadequate adherence to the behavioral support intervention is a major protocol deviation that results in an inconclusive trial. Researchers conducting behavior change trials should resist the temptation of interpreting a positive, negative, or inconclusive behavior change trial that improved secondary health outcomes as a positive effectiveness trial.

**Figure 3:**
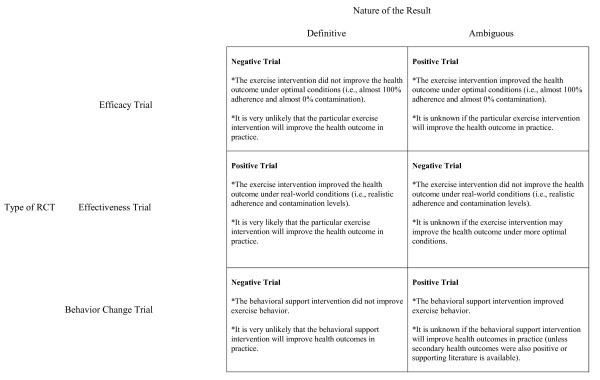
**Nature of the trial result produced by three types of exercise trials**.

The types of exercise RCTs also vary in regard to the nature of the definitive result that each trial produces (Figure 3) [7]. For efficacy trials, the definitive result is a negative trial. In that case, it is certain that the exercise intervention has no effect on the health outcome. A positive efficacy trial is an ambiguous finding because it is unknown if the health outcome can be achieved in practice. The converse is true for effectiveness trials [7]. A positive effectiveness trial is the definitive result because the trial demonstrates that the health outcome can be improved under "real-world" conditions although additional steps toward dissemination may be required [2]. A negative effectiveness trial may be ambiguous because it is unclear if a positive effect on the health outcome could have been achieved under more favorable conditions [7]. Finally, a negative behavior change trial is a definitive result because it is clear that the behavioral support intervention would not improve any health outcomes in practice. A positive behavior change trial may be ambiguous because it is unclear if it will actually improve health outcomes in practice (unless important secondary health outcomes were also improved in the trial or there is compelling evidence of the effect of such behavior change from previous studies).

## Discussion

The purpose of this paper was to offer some conceptual and methodological distinctions between health outcome trials (efficacy and effectiveness) and behavior change trials. It was argued that these trials differ in terms of many important attributes and design issues. Several issues raised in this paper warrant further discussion.

One important issue is whether it is possible to conduct both a behavior change trial and a health outcome trial as part of the same trial. The difficulty with this approach is that it requires multiple primary outcomes, which is not recommended by CONSORT [1]. Multiple primary outcomes may lead to internally inconsistent decisions because the selection of a primary outcome drives all other methodological decisions in the trial [1,7,8]. For example, an exercise RCT may select exercise behavior, weight loss, and depression as its primary outcomes. In this trial, would the eligibility criteria include only nonexercisers who are obese and depressed? Or would it allow current exercisers who are obese and depressed, or obese people who are not depressed and depressed people who are not obese? What exercise intervention would be selected? Perhaps a very high volume of exercise might be best for weight loss but not for depression. Perhaps yoga would be great for depression but not for weight loss. What about the selection of gold standard measures given limited resources? Should the researchers spend significant money on the gold standard measures of exercise behavior (e.g., direct observation, accelerometers) or obesity (e.g., DEXA scans, CT scans) or depression (e.g., clinical interviews)? Will the sample size be determined by expected changes in exercise, obesity, or depression? What will the comparison group receive? Should the comparison group be asked not to exercise? How will the intervention be interpreted? Will it be successful if people exercise but are still obese and depressed? Is it successful if they lose weight but are still depressed, or are less depressed but still obese? Trial design issues are often difficult to resolve when there are competing primary outcomes.

It seems reasonable for a behavior change trial to have a secondary purpose of documenting changes in health outcomes but the trial would still have been conceptualized and designed as a behavior change trial with all the important trial decisions based on this purpose. It does not seem reasonable, however, for a health outcome trial to have a secondary purpose of testing a behavior support intervention because the former is actually contingent on the latter. That is, the health outcome trial is unable to answer its primary question if behavior change does not occur (i.e., it becomes an inconclusive trial). If behavior change is uncertain in a health outcome trial, it seems the best approach would be to conduct a feasibility study demonstrating that behavior change is highly likely before moving to the health outcome trial.

Another interesting issue is whether it is possible to make the same conceptual distinction between efficacy and effectiveness for behavior change trials as it is for health outcome trials. In the present paper, behavior change trials have been largely conceptualized as effectiveness trials by default. Perhaps it would have been more prudent to develop a 2 × 2 typology of trial outcome (behavior change versus health outcome) by trial attitude (efficacy versus effectiveness). In considering this option, it does not seem helpful to conduct efficacy-oriented behavior change trials because the primary purpose of such trials is usually to translate health outcome research into practice. Moreover, it does not seem to make sense for behavior change trials to only select highly motivated participants or to use a behavioral support intervention that is not feasible for practice when the goal is to disseminate the exercise research to as many people as possible in the target population. It would seem that the only reason for incorporating a behavioral support intervention that is not feasible in practice is because the goal of the trial is to establish the efficacy of the health intervention. Having said that, the utility of distinguishing between efficacy and effectiveness behavior change trials may warrant further discussion.

It is also critical to note that what is feasible to be put into practice varies dramatically from context to context [7,8]. For example, supervised exercise protocols delivered by highly qualified personnel may indeed be readily transferable into practice in certain clinical settings with dedicated space, sophisticated equipment, and highly qualified personnel (e.g., cardiac rehabilitation unit). Similarly, supervised exercise protocols may even be feasible to deliver in many community-based settings such as public and private fitness and recreational centers that possess the necessary equipment and qualified staff. Conversely, supervised exercise protocols may not be readily transferable into practice in rural communities or in public health settings because of the lack of facilities, personnel, and limited funding. The key point is that what is considered feasible to be translated into exercise practice varies with the practice setting. Consequently, exercise researchers conducting effectiveness trials should make explicit the intended practice setting for their intervention and design their trial accordingly [8].

Another important issue is the order of conducting the different types of exercise RCTs. As noted earlier, it is generally argued that efficacy trials should precede effectiveness trials [4]. That is, once the efficacy of an intervention is proven, then it needs to be tested for its effectiveness. In exercise research, efficacy trials followed by an attempt to put the positive results into practice without the demonstration of effectiveness may fail in settings that cannot deliver supervised exercise interventions or provide extensive behavioral support interventions (e.g., rural, public health). One might reasonably argue that, in practice settings where supervised exercise or extensive behavioral support interventions are not likely feasible, it may be more productive to begin with contemporary effectiveness trials that test exercise interventions that are feasible even though they may not have proven efficacy. Thus, in exercise research, effectiveness trials might also emanate from positive behavior change trials with proven feasibility in a given practice setting.

This paper provides a first attempt to highlight some conceptual and methodological issues that have arisen with the incorporation of modern behavioral support interventions into many exercise trials. It was a difficult paper to write because of the complexity of this topic and the diversity of exercise research. There are significant limitations in this paper. One limitation is that the paper does not attempt to integrate the distinction between health outcome trials and behavior change trials into other existing research typologies beyond the common efficacy-effectiveness distinction (e.g., Phase I-IV trials, translational trials, dissemination trials, comparative effectiveness trials, research phases, etc.). A second limitation is that every exercise trial is unique and some of the general observations made in this paper may not apply to individual exercise trials. A third limitation is that there does not appear to be a clear distinction between efficacy and effectiveness trials because it is the individual elements of a trial that vary along the efficacy-effectiveness continuum and researchers might intentionally, and appropriately, mix efficacy and effectiveness attributes [8]. Another limitation is that the paper does not address research designs beyond the traditional RCT. Finally, it is unclear if the issues raised in this paper apply to other health behavior interventions (e.g., nutrition) or to interventions that target multiple health behaviors.

In summary, exercise RCTs are growing in sophistication and complexity because of the desire to test longer exercise interventions, unsupervised exercise interventions, and higher volume exercise interventions. These complexities have led to the incorporation of behavioral support interventions into many exercise RCTs and has sometimes confused the primary purpose of the trial. Researchers conducting exercise RCTs are encouraged to consider the nature of their trial in terms of its health outcome versus behavior change focus and its position on the efficacy versus effectiveness continuum. Clarity in the goals of the trial may improve the trial methods and result in better quality trials more likely to inform clinical, community, and public health exercise interventions.

## Competing interests

The authors declare that they have no competing interests.
